# Trends, underlying-cause structure, and penetration of end-stage kidney disease involvement in U.S. mortality, 1999–2023: a multiple-cause-of-death analysis

**DOI:** 10.3389/fpubh.2026.1831145

**Published:** 2026-06-19

**Authors:** Kaide Xia, Junwen Wang, Zefa Meng, Jingwen Yan

**Affiliations:** 1 Guiyang Children's Hospital, The Maternal and Child Health Care Hospital of Guizhou Medical University, Guiyang, China; 2 Department of Psychosomatic Medicine, The Second People’s Hospital of Guiyang, Guiyang, China; 3 Department of Nephrology, Zhejiang Provincial People’s Hospital Bijie Hospital (The First People’s Hospital of Bijie), Bijie, China

**Keywords:** CDC WONDER, end-stage kidney disease (ESKD), epidemiology, health disparities, mortality trends, Multiple Cause of Death

## Abstract

**Background:**

End-stage kidney disease (ESKD) is often listed as a contributing rather than underlying cause of death, potentially understating its burden in underlying-cause surveillance. Using U.S. multiple-cause-of-death data, we evaluated trends, underlying-cause pathways, ESKD “penetration” into cause-specific mortality, and drivers of change in ESKD-involved deaths.

**Methods:**

Using CDC WONDER Multiple Cause of Death data (1999–2023), we identified ESKD-involved deaths among adults aged ≥25 years and calculated age-adjusted mortality rates (AAMR; 2000 U.S. standard). Joinpoint regression was performed using a log-linear model with a maximum of two joinpoints. Model selection was conducted using the Joinpoint Regression Program’s default permutation-test procedure and grid-search settings. Average annual percent change (AAPC) and 95% CIs were estimated over the prespecified analysis intervals. For race-specific analyses, AAPCs were restricted to 1999–2020 bridged-race files; for urbanization analyses, AAPCs were restricted to 1999–2020. Underlying-cause composition was assessed using ICD-10 chapters and the NCHS 113 selected-cause list. ESKD penetration (*P_g,t_*) was estimated within each underlying-cause group, and a symmetric two-factor decomposition separated changes in ESKD-involved deaths into scale versus penetration components, comparing pre–COVID-19 and COVID-19-era periods.

**Results:**

ESKD-involved AAMR increased from 14.51 (95% CI 14.33–14.69) per 100,000 in 1999 to 18.74 (18.58–18.91) in 2023 (AAPC 1.33%, 0.61–2.10). Increases were faster in males than females (2.06% vs. 1.07) and in adults aged ≥65 vs. 25–64 years (1.81% vs. 0.73). In race-specific analyses restricted to the 1999–2020 bridged-race files, Black individuals had the highest absolute mortality, whereas White individuals showed a significant increase. The underlying-cause structure shifted, with genitourinary causes becoming more prominent. *P_g,t_* varied widely by cause and increased for selected categories, indicating growing ESKD co-occurrence with specific fatal pathways. Decomposition showed heterogeneous scale versus penetration contributions across causes and period-specific differences.

**Conclusion:**

ESKD involvement in U.S. mortality rose from 1999 to 2023 with marked subgroup inequities and shifts in underlying-cause pathways. Monitoring ESKD as a contributing cause, together with cause-structure and scale–penetration analyses, provides information beyond underlying-cause surveillance to support integrated prevention addressing CKD progression and its cardiometabolic and infectious complications.

## Introduction

End-stage kidney disease (ESKD), the terminal stage of chronic kidney disease, confers a substantial excess risk of cardiovascular events, infection, metabolic complications, and premature death (1–5). Despite its severity, ESKD is seldom recorded as the underlying cause of death on death certificates (6–8). It more frequently appears as a contributing condition alongside proximate terminal diagnoses such as cardiovascular disease, diabetes-related complications, sepsis, or respiratory failure (9, 10). Consequently, surveillance systems relying solely on the underlying cause of death may substantially underestimate the population-level mortality burden attributable to ESKD and obscure its role in various fatal disease pathways (6, 7, 11).

Multiple-cause-of-death data offer a complementary perspective by capturing all conditions listed on the death certificate, thereby enabling an assessment of ESKD as part of the causal sequence leading to death (12). This “any-mention” framework is particularly valuable for chronic conditions that amplify mortality risk across multiple etiologies (13, 14). National evidence describing long-term trends in ESKD-involved mortality, the evolving composition of underlying causes among these deaths, and the extent of ESKD’s embedding within deaths from non-kidney causes remains limited, however (15). These knowledge gaps are especially salient given changing cardiometabolic risk profiles, secular shifts in dialysis populations and treatment patterns, and the major disruptions to U.S. mortality during the COVID-19 era (16–18).

Beyond describing the overall burden, assessing disparities in ESKD-involved mortality across population subgroups is critical (19, 20). Social and structural determinants, comorbid disease patterns, and inequities in access to prevention, nephrology care, dialysis modalities, and transplantation shape the incidence and progression of ESKD (21–23). How ESKD involvement on death certificates has changed over time across demographic and geographic strata—and whether trend patterns differ by subgroup—has not been comprehensively quantified using national multiple-cause mortality data spanning more than two decades (20, 24).

To address these gaps, we conducted a national study of U.S. residents aged ≥25 years from 1999 through 2023 using the CDC WONDER Multiple Cause of Death database. Our analysis had four aims: to quantify temporal trends and joinpoints in ESKD-involved age-adjusted mortality (25); to characterize changes over time in the underlying-cause composition of these deaths using International Classification of Diseases, 10th Revision (ICD-10) chapter and 113 selected-cause groupings; to estimate the ESKD involvement proportion, *P_g,t_*, within each underlying-cause group as a measure of ESKD “penetration” into cause-specific mortality (12); and to decompose changes in ESKD-involved deaths into contributions from changes in the underlying-cause mortality base (scale effect) versus changes in ESKD involvement (penetration effect). By integrating burden, structure, and penetration metrics, this study provides a comprehensive view of how ESKD’s role on death certificates has evolved and which underlying-cause pathways contribute most to the changing ESKD-involved mortality landscape in the United States.

## Methods

### Data source and study population

We obtained mortality data for 1999–2023 from the Centers for Disease Control and Prevention (CDC) WONDER Multiple Cause of Death database (11). The study population comprised all U.S. residents aged 25 years or older. We restricted the study population to adults aged ≥25 years because ESKD-involved deaths are rare in younger individuals, more vulnerable to sparse-cell suppression in CDC WONDER, and may reflect different pediatric, congenital, or early-onset etiologies. Due to a transition in CDC race reporting standards, we analyzed two datasets separately: the Bridged-Race files for 1999–2020 and the Single-Race files for 2021–2023. Consequently, race-specific findings are interpreted within each reporting framework and are not combined into a single continuous series. We treated suppressed cells in CDC WONDER as missing data. Estimates derived from small numbers were interpreted with caution, in accordance with National Center for Health Statistics (NCHS) standards for suppressing unreliable estimates.

### Case definition and cause-of-death classification

We defined ESKD-involved mortality as deaths with end-stage kidney disease recorded anywhere on the death certificate, identified using the ICD-10 mortality codes available in CDC WONDER (N18.0 or N18.5) in any multiple-cause-of-death field (26). CDC WONDER mortality data are coded using the WHO ICD-10 mortality classification rather than ICD-10-CM; accordingly, ICD-10-CM codes such as N18.6 are not available in this database. Because the clinical ICD-10-CM code N18.6 is not available in CDC WONDER mortality files, we used the available mortality-code categories most closely corresponding to ESKD-related conditions. To evaluate the temporal behavior of these codes and the apparent discontinuity around 2011–2013, we conducted a code-specific verification analysis by querying N18.0 and N18.5 separately by year among adults aged ≥25 years, using the same multiple-cause mortality settings and the 2000 U.S. standard population as in the main analysis. The underlying cause of death (UCD) was classified using two hierarchical schemes: ICD-10 chapters for high-level structural analysis and cross-year comparability, and the NCHS 113 Selected Causes list for more granular etiologic characterization. For clarity in visualizations, lengthy ICD chapter labels were abbreviated; for example, “Endocrine, nutritional and metabolic diseases” became “Endocrine/Metab.” A full mapping of abbreviations is provided in Supplementary Table S1.

### Statistical analysis

Age-adjusted mortality rates (AAMRs) per 100,000 population, standardized to the 2000 U.S. standard population, were obtained directly from CDC WONDER outputs with corresponding 95% confidence intervals (27, 28). Temporal trends in these rates were analyzed with the National Cancer Institute Joinpoint Regression Program (Version 5.4.0), which fits log-linear models to estimate both segment-specific and full-period average annual percent changes (APC and AAPC) (29). Joinpoint regression was performed using a log-linear model with a maximum of two joinpoints. Model selection was conducted using the Joinpoint Regression Program’s default permutation-test procedure and grid-search settings. For race-specific analyses, AAPCs were restricted to the 1999–2020 bridged-race files; for urbanization analyses, AAPCs were also estimated for 1999–2020. To characterize shifts in the underlying-cause structure among ESKD-involved deaths, we determined the annual proportional composition of deaths attributable to each underlying cause of death (UCD) group. A Top-9 + Other strategy was employed, retaining the nine underlying-cause groups with the largest cumulative deaths across 1999–2023 and aggregating all remaining causes into an “Other” category. This strategy was chosen *a priori* to create a readable ten-category display while retaining the leading ICD-10 chapter groups by cumulative ESKD-involved deaths. The annual proportional composition was visualized using 100% stacked area plots. Changes in cause ranking between 1999 and 2023 were further illustrated with rank-shift plots based on cause-specific ESKD-involved death counts.

We quantified disease-specific ESKD “penetration” by estimating the ESKD involvement proportion *P_g,t_*, defined as the proportion of deaths within a given underlying-cause group g and year t in which ESKD was also recorded anywhere on the death certificate. In practical terms, *P_g,t_* captures the within-cause involvement of ESKD rather than the absolute number of ESKD-involved deaths:


$$P_{g,t}=\frac{\begin{array}{l} \text{Deaths with underlying cause}\;g\;\text{and ESKD}\;\\ \text{mentioned anywhere}\;\mathrm{on}\;\text{the death certificate} \end{array}}{\mathrm{All}\;\text{deaths with underlying cause}\;g}$$

The numerator was derived from CDC WONDER queries restricted to deaths involving ESKD (any mention of N18.0/N18.5), grouped by year and underlying-cause category, while the denominator came from parallel queries for all deaths without an ESKD restriction, grouped identically. We summarized the temporal patterns in *P_g,t_* using heatmaps and complementary faceted time-series plots. To improve heatmap interpretability, cause groups with insufficient temporal coverage—specifically, those with a high proportion of missing *P_g,t_* values due to suppressed or unavailable counts—were excluded according to a prespecified threshold. For *P_g,t_* heatmaps and decomposition analyses, ICD-10 chapter groups were included if *P_g,t_* estimates were available for at least 90% of years in the corresponding analysis window. Suppressed or unreliable CDC WONDER cells were treated as missing rather than zero, except in the code-specific verification analysis, where zero-count outputs were explicitly requested to evaluate temporal code availability.

For each underlying-cause group g and year *t*, we expressed the number of ESKD-involved deaths as *E_g,t_ = D_g,t_ × P_g,t_*, where *D_g,t_* denotes the total deaths with underlying cause *g* (denominator from all-deaths queries) and *P_g,t_* denotes the ESKD involvement proportion. To quantify drivers of change in ESKD-involved deaths between two time points (*t_0_, t_1_*), we applied a symmetric two-factor decomposition [Kitagawa/Das Gupta formulation (30, 31)] to partition *ΔE_g_ = E_g,t1_ − E_g,t0_* into a scale effect attributable to changes in the underlying-cause mortality base and a penetration effect attributable to changes in ESKD involvement:


$$\Delta E_{g}^{\text{scale}}=(D_{g,t1}-D_{g,t0})\times \frac{P_{g,t0}+P_{g,t1}}{2}$$

$$\Delta E_{g}^{\text{penetration}}=(P_{g,t1}-P_{g,t0})\times \frac{D_{g,t0}+D_{g,t1}}{2}$$

By construction, $\Delta E_{g}\;$ = $\Delta E_{g}^{\text{scale}}+\Delta E_{g}^{\text{penetration}}$ , apart from negligible numerical rounding error.

We conducted decomposition for the full interval (1999–2023) and, to assess potential disruption during the COVID-19 era, repeated the analysis for 1999–2019 and 2019–2023. Cause groups with insufficient *P_g,t_* temporal coverage due to suppressed or unavailable counts were excluded using the same prespecified coverage criterion applied in *P_g,t_* visualizations. Results were summarized using stacked contribution plots, a scale–penetration scatter plot, and 100% share plots. Statistical significance for trend estimates was assessed using a two-sided *α* of 0.05 with 95% confidence intervals (CIs); decomposition results were interpreted descriptively.

## Results

### Temporal trends and demographic disparities in ESKD-involved mortality

**Figure 1:**
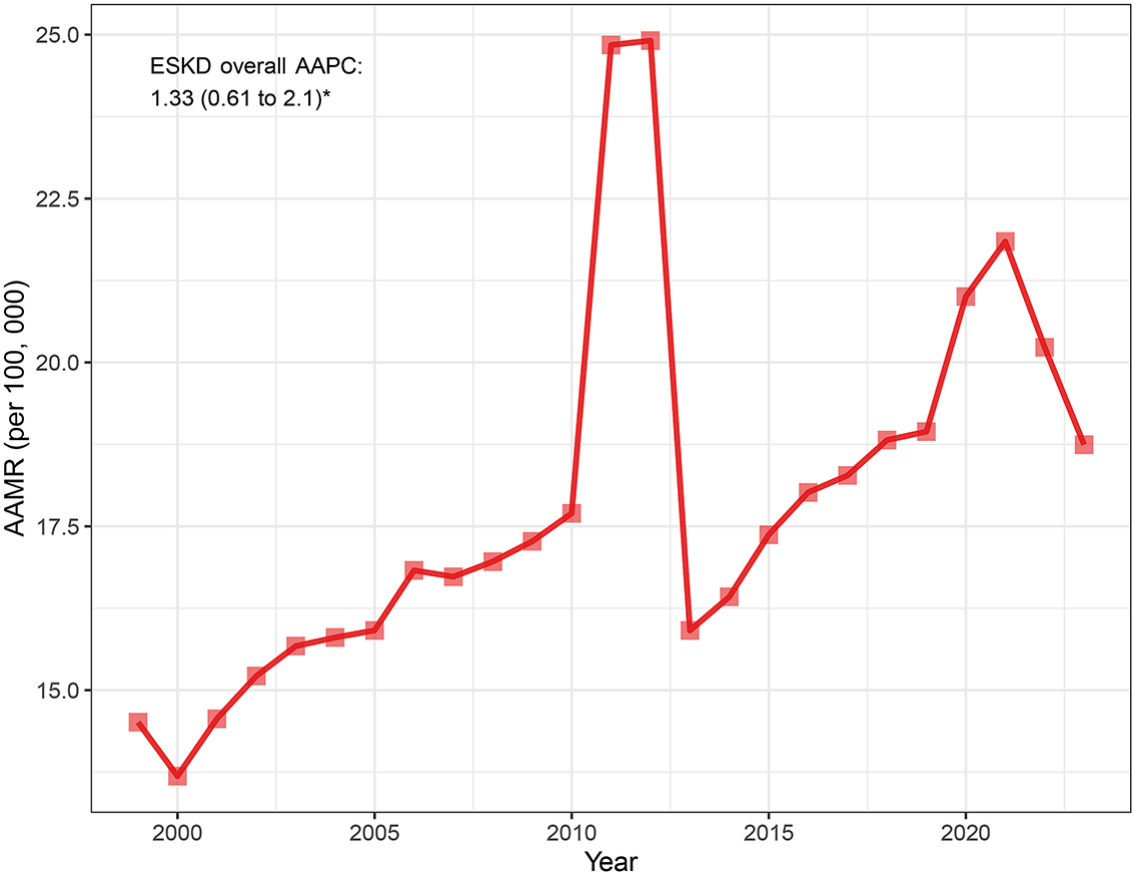
National trends in ESKD-involved AAMR, 1999–2023. AAMR, age-adjusted mortality rate; ESKD, end-stage kidney disease. Line chart showing the U.S. AAMR for ESKD-involved deaths among adults aged ≥25 years from 1999 to 2023. The rate increases overall with non-linear fluctuations, including higher levels in the early 2010s and during 2020–2023. The apparent elevation around 2011–2013 should be interpreted cautiously in light of the code-specific verification analysis shown in Supplementary Table S2.

From 1999 to 2023, ESKD-involved mortality in U.S. adults increased substantially. The AAMR rose from 14.51 (95% CI, 14.33–14.69) per 100,000 population in 1999 to 18.74 (18.58–18.91) in 2023, corresponding to an overall AAPC of 1.33% (0.61 to 2.10) (Table 1; Figure 1). Joinpoint analysis indicated a non-linear pattern, with relatively modest changes in the early period followed by a pronounced increase after approximately 2011 and a clear elevation in 2020–2023 (Figure 1). A code-specific verification analysis showed that N18.0 was observed during 1999–2010 but not after 2010, whereas N18.5 was not observed during 1999–2010 and became the only observed ESKD-related code from 2011 onward, including 2021–2023 in the single-race files (Supplementary Table S2). Therefore, the apparent fluctuation around 2011–2013 was interpreted as reflecting a coding transition in CDC WONDER mortality data rather than solely a biological change in ESKD-involved mortality.

**Table 1:** Temporal trends in ESKD-involved age-adjusted mortality among U.S. adults.

Categories	1999		2023 or latest available year		AAPC (95% CI)
	Counts	AAMR (95% CI)	Counts	AAMR (95% CI)	
Overall	25,738	14.51 (14.33, 14.69)	51,773	18.74 (18.58, 18.91)	1.33 (0.61 to 2.1)^*^
Sex					
Female	12,717	12.67 (12.45, 12.89)	23,130	15.29 (15.09, 15.49)	1.07 (0.37 to 1.81)^*^
Male	13,021	17.38 (17.08, 17.69)	28,643	23.18 (22.90, 23.45)	2.06 (1.16 to 2.97)^*^
Age					
25–64	8,680	6.10 (5.97, 6.23)	12,956	6.43 (6.31, 6.54)	0.73 (−0.02 to 1.53)
≥65	17,058	49.15 (48.41, 49.89)	38,817	69.45 (68.75, 70.14)	1.81 (0.89 to 2.92)^*^
Race^a^					
Black	8,153	50.22 (49.11, 51.32)	14,608	51.94 (51.07, 52.80)	−0.06 (−0.97 to 0.96)
Other	1,058	20.27 (18.99, 21.56)	3,306	20.08 (19.39, 20.78)	0.68 (−0.22 to 2.07)
White	16,527	10.60 (10.44, 10.77)	35,181	17.10 (16.93, 17.28)	2.6 (1.6 to 3.66)^*^
Urbanization^a^					
Metropolitan	21,756	15.04 (14.84, 15.24)	47,198	21.08 (20.89, 21.28)	2.00 (0.9 to 3.16)^*^
Nonmetropolitan	3,982	12.24 (11.86, 12.62)	8,867	20.82 (20.37, 21.27)	2.26 (1.39 to 3.26)^*^
Census region					
Northeast	5,217	14.16 (13.77, 14.54)	8,112	15.89 (15.54, 16.24)	0.84 (−0.04 to 1.81)
Midwest	4,746	11.38 (11.06, 11.71)	10,951	18.89 (18.53, 19.25)	2.61 (1.66 to 3.77)^*^
South	11,369	18.04 (17.71, 18.38)	22,087	21.00 (20.72, 21.28)	1.02 (0.38 to 1.71)^*^
West	4,406	12.28 (11.92, 12.65)	10,623	17.03 (16.70, 17.36)	2.29 (1.46 to 3.46)^*^

a Race-specific AAPCs were estimated using the 1999–2020 bridged-race files only.

Single-race estimates for 2021–2023 were summarized descriptively in Supplementary Table S3 and were not combined with bridged-race estimates to derive continuous race-specific AAPCs. Urbanization-stratified AAPCs were also estimated for 1999–2020. *Indicates that the AAPC was statistically significantly different from zero at *p* < 0.05. AAPC, average annual percent change; AAMR, age-adjusted mortality rate; CI, confidence interval; ESKD, end-stage kidney disease.

Stratified analyses demonstrated marked heterogeneity across population subgroups (Table 1; Supplementary Figure S1). ESKD-involved mortality increased more rapidly among males than females (AAPC: 2.06% vs. 1.07%) (Supplementary Figure S1A). By race, analyses restricted to the 1999–2020 bridged-race files showed that Black individuals had the highest absolute ESKD-involved AAMR, while only White individuals had a significant increasing trend (AAPC: 2.60, 95% CI: 1.60 to 3.66; Supplementary Figure S1B). Race-specific Joinpoint analyses were not extended beyond 2020 because of the transition from bridged-race to single-race files; 2021–2023 single-race estimates are presented descriptively in Supplementary Table S3. Older adults (≥65 years) experienced a steeper increase than adults aged 25–64 years (AAPC: 1.81% vs. 0.73%) (Supplementary Figure S1C). Geographic and urban–rural patterns were also observed: census-region trends varied (Supplementary Figure S2), and urbanicity analyses (available through 2020) showed increasing ESKD-involved mortality in both metropolitan and nonmetropolitan areas (Supplementary Figure S1D).

### Shifts in the underlying-cause composition of ESKD-involved deaths

**Figure 2:**
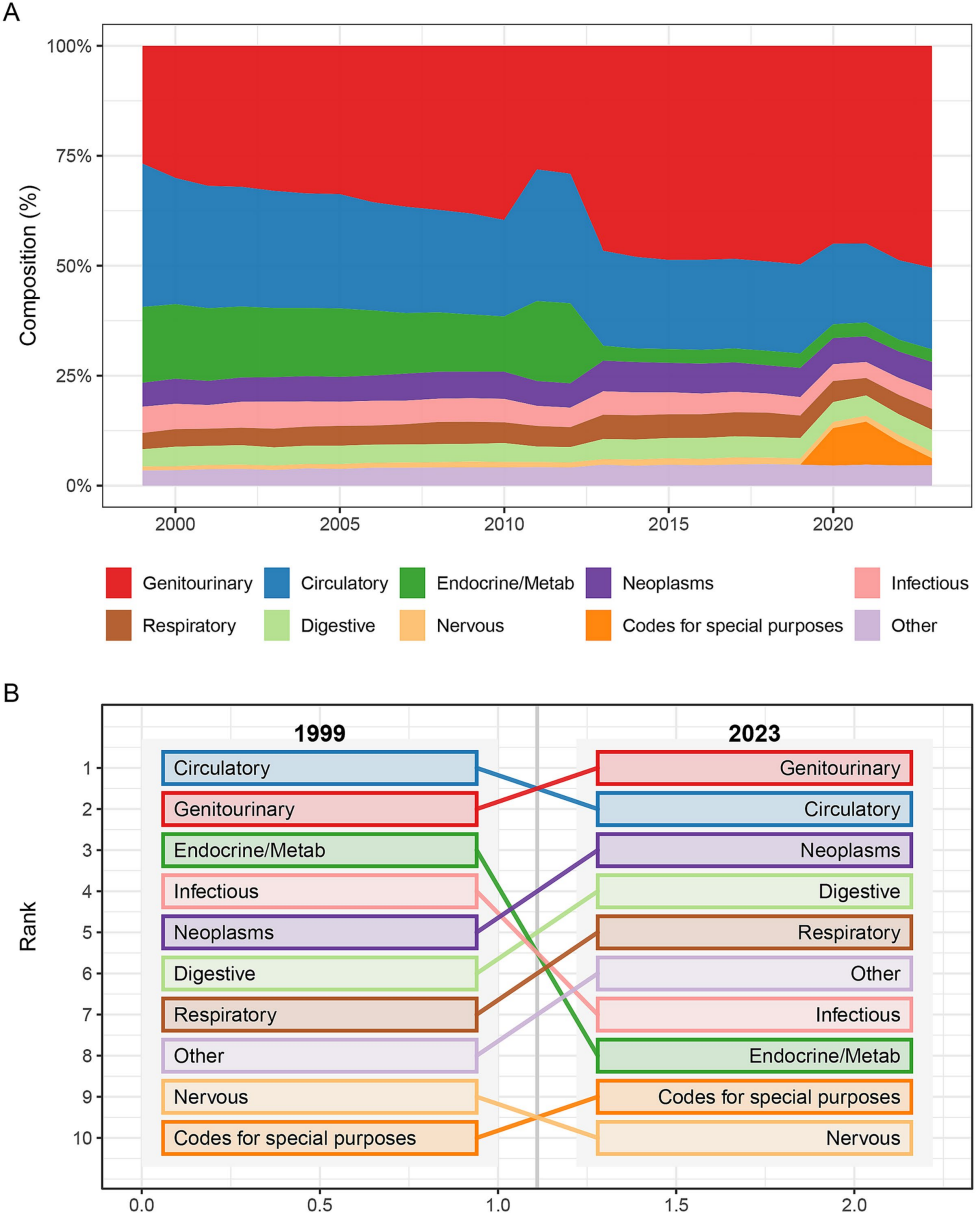
Underlying-cause composition of ESKD-involved deaths, 1999–2023. **(A)** A 100% stacked area plot showing shifting proportions of the top nine ICD-10 chapter categories plus “Other” over time. **(B)** A rank-shift diagram comparing the ordering of leading categories between 1999 and 2023. ESKD, end-stage kidney disease.

**Figure 3:**
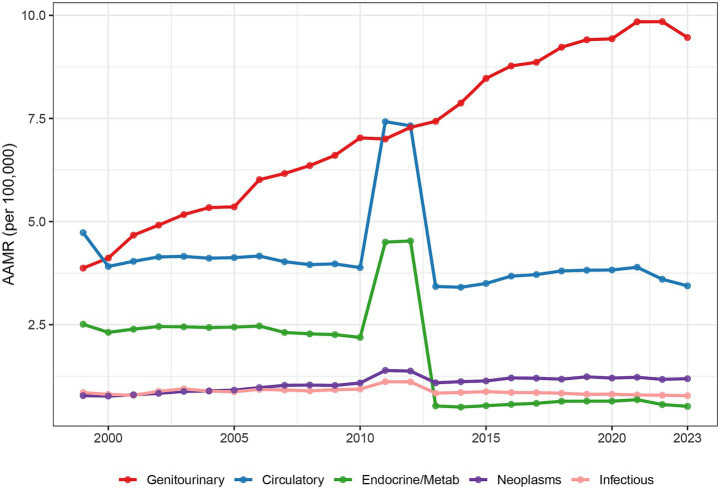
Trends in the leading underlying-cause categories among ESKD-involved deaths, 1999–2023. ESKD, end-stage kidney disease. Multi-line chart showing age-adjusted mortality trends from 1999 to 2023 for the five leading ICD-10 chapter–defined underlying-cause categories among ESKD-involved deaths. The lines illustrate distinct temporal patterns across causes, with some categories increasing more rapidly than others over the study period.

The underlying-cause structure of ESKD-involved deaths changed substantially between 1999 and 2023 (Figure 2). In the late 1990s, circulatory diseases represented the largest proportion of these deaths; however, the composition shifted over time, with diseases of the genitourinary system becoming the leading ICD chapter by the end of the study period. Endocrine, nutritional, and metabolic diseases also grew in prominence, whereas other major categories, such as neoplasms and respiratory diseases, contributed smaller or relatively stable shares. Rank-shift visualization further underscored a reordering of the leading categories between 1999 and 2023, confirming that the hierarchy of underlying causes was not static (Figure 2B). Trends in the leading ICD-10 chapter-defined underlying-cause categories further showed distinct time-varying patterns over the study period (Figure 3). Trends based on 113 selected causes provided higher-resolution corroboration of these shifts, demonstrating that kidney-related and major cardiovascular causes remained dominant contributors, with several causes exhibiting distinct time-varying patterns (Supplementary Figures S3, S4).

### ESKD involvement proportion (*P_g,t_*) across underlying-cause categories

**Figure 4:**
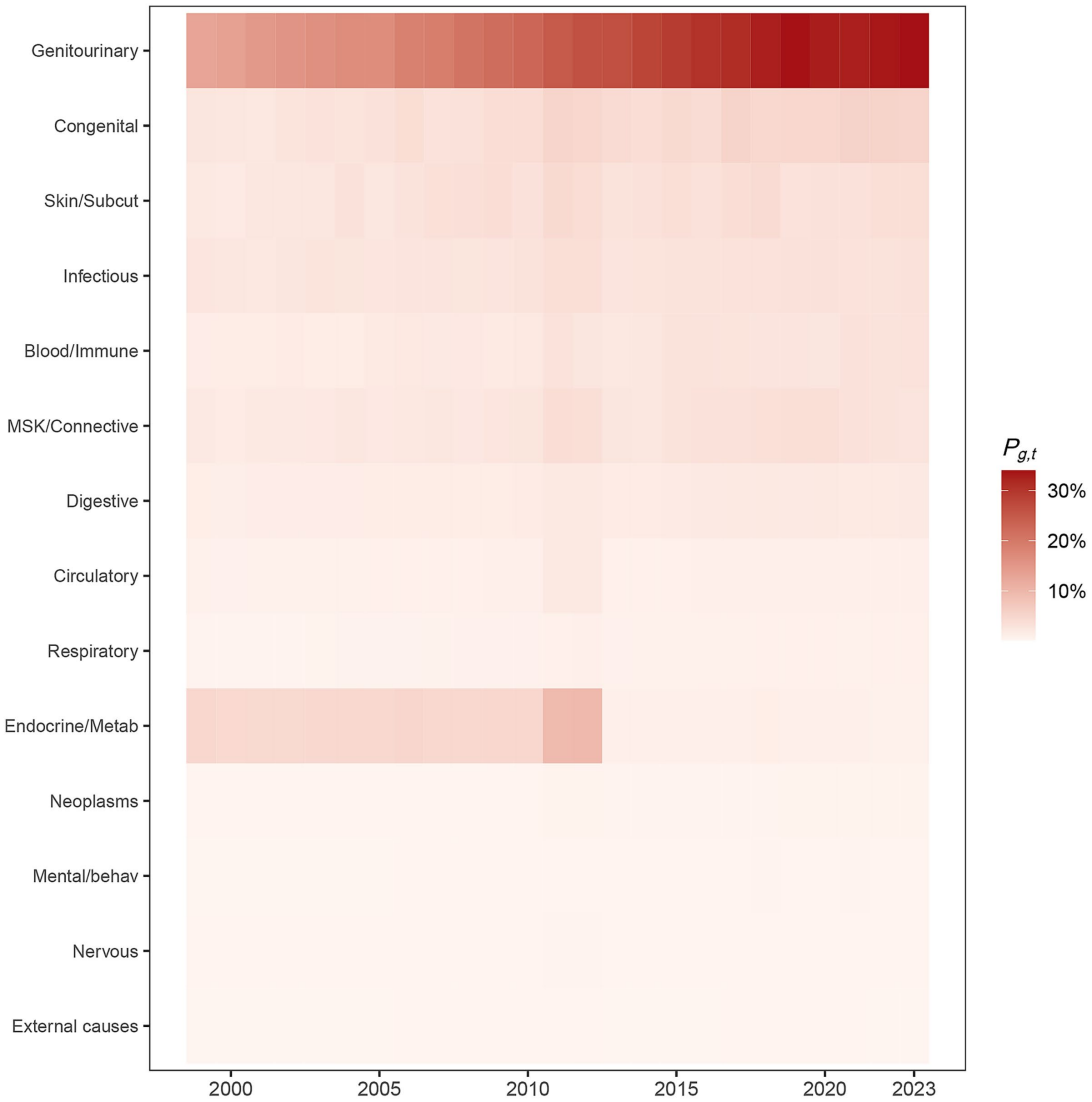
ESKD involvement proportion (*P_g,t_*) by underlying-cause category, 1999–2023. ESKD, end-stage kidney disease. *P_g,t_* was defined as the proportion of deaths within underlying-cause group *g* in year *t* in which ESKD was also recorded anywhere on the death certificate. Heatmap showing the ESKD involvement proportion (*P_g,t_*) by ICD-10 chapter–defined underlying cause from 1999 to 2023. Each tile represents a year–cause combination, with darker shading indicating higher *P_g,t_*. *P_g,t_* is highest for genitourinary causes and varies across categories and over time. Gray or blank tiles indicate suppressed or unavailable estimates and should not be interpreted as zero *P_g,t_.*

To assess the “penetration” of ESKD into deaths from specific underlying-cause categories, we estimated *P_g,t_*—the proportion of deaths within each ICD chapter that also mentioned ESKD—across 1999–2023. Temporal coverage of *P_g,t_* estimates varied across ICD-10 chapters; therefore, *P_g,t_* heatmaps and decomposition analyses were restricted to chapters meeting the prespecified ≥90% temporal coverage criterion (Supplementary Table S4). *P_g,t_* varied substantially by underlying cause and changed over time. Diseases of the genitourinary system consistently exhibited the highest *Pg,t*, with progressively increasing values over time. Endocrine/metabolic causes also showed a discernible upward pattern in *P_g,t_*, indicating that ESKD became increasingly common as a contributing condition among deaths attributed to these causes. In contrast, *P_g,t_* for circulatory diseases remained comparatively low and stable, and neoplasms showed persistently minimal ESKD involvement (Figure 4; Supplementary Figure S5). Results were visually consistent across the heatmap representation (year × ICD chapter) and the faceted trend-line display, which together emphasize both the cross-cause heterogeneity and the temporal evolution of ESKD involvement (Figure 4; Supplementary Figure S5). Abbreviations used for ICD chapter labels are provided in the supplementary mapping table (Supplementary Table S1).

### Drivers of change in ESKD-involved deaths: scale versus penetration effects

Decomposition analyses indicated that the increase in ESKD-involved deaths was not driven uniformly across underlying-cause categories. In stacked contribution analyses for 1999–2023, diseases of the genitourinary system accounted for the largest absolute increase in ESKD-involved deaths, with contributions arising from both an increase in underlying-cause deaths (scale) and an increase in ESKD involvement proportion (penetration) (Supplementary Figure S6). Across other major ICD chapters, the relative importance of penetration versus scale varied, and in some categories the two components partially offset each other, indicating that changes in ESKD involvement proportions did not always track changes in the underlying-cause mortality base (Supplementary Figures S7, S8). The scale–penetration scatter further highlighted cross-cause heterogeneity: some chapters clustered in the quadrant with concurrent positive scale and penetration contributions, whereas others exhibited discordant patterns (e.g., positive penetration but negative scale or vice versa), underscoring that ESKD’s embedding within cause-specific mortality pathways evolved differently by disease category (Supplementary Figure S7). In sensitivity analyses stratified by period, the driver profile differed between 1999–2019 and 2019–2023. During 2019–2023, several categories showed comparatively larger scale contributions—consistent with broad shifts in mortality during the COVID-19 era—while genitourinary causes remained a dominant contributor overall (Supplementary Figure S9). These period-specific patterns suggest that pandemic-era changes in the underlying-cause mortality base contributed to short-term changes in ESKD-involved deaths.

## Discussion

Using national multiple-cause-of-death data, this study provides a longitudinal characterization of ESKD-involved mortality among U.S. adults from 1999 through 2023. Three key findings emerged. First, ESKD-involved age-adjusted mortality increased over time with clear non-linearity, including acceleration after the early 2010s and an additional elevation during 2020–2023, coinciding with the COVID-19 era. Second, the underlying-cause landscape among ESKD-involved deaths was dynamic: both the composition and the ranking of leading underlying causes shifted substantially across decades, and cause-specific trajectories were heterogeneous. Third, ESKD involvement proportion (*P_g,t_*)—our measure of ESKD “penetration” into cause-specific mortality—varied markedly by underlying cause and changed over time, suggesting that ESKD increasingly co-occurs with selected fatal pathways rather than appearing solely as a terminal kidney diagnosis.

These findings underscore an important limitation of conventional mortality surveillance that relies primarily on the underlying cause of death (32, 33). Because ESKD is frequently recorded as a contributing condition in deaths ultimately attributed to cardiovascular disease, diabetes/metabolic disorders, infections, or other chronic illnesses, underlying-cause-only approaches may underestimate ESKD’s population impact and obscure how ESKD is embedded within broader mortality pathways (32, 34–36). By applying an any-mention framework, we capture ESKD as part of the certified causal chain and show that its role on death certificates has evolved over time. The observed reconfiguration of underlying causes among ESKD-involved deaths likely reflects a combination of changing cardiometabolic risk profiles, secular shifts in dialysis populations and treatment patterns, and evolving competing risks that influence which proximate conditions are selected as the underlying cause (33). In parallel, the *P_g,t_* patterns indicate that ESKD is not uniformly distributed across diseases: it is highly concentrated within genitourinary causes and increasingly present within endocrine/metabolic deaths, while remaining comparatively uncommon in deaths attributed to neoplasms and other categories—patterns consistent with distinct pathophysiologic links and care-delivery interfaces between ESKD and different terminal conditions (37, 38). However, *P_g,t_* should not be interpreted as a causal attribution measure. Rather, it reflects the proportion of deaths within a given underlying-cause category in which ESKD was also recorded anywhere on the death certificate. Changes in *P_g,t_* may reflect true changes in comorbidity patterns and disease pathways, but they may also be influenced by death-certification practices, coding conventions, and documentation of chronic comorbidities.

The stratified analyses further suggest substantial demographic and geographic inequities in ESKD-involved mortality (39–41). These differences plausibly reflect variation in upstream CKD incidence and progression, comorbidity burden (particularly hypertension and diabetes), access to preventive care and nephrology services, dialysis modality availability, transplantation access, and broader structural determinants of health (39, 42, 43). From a prevention and policy perspective, the results reinforce that ESKD involvement is not merely a nephrology endpoint; it is intertwined with cardiometabolic and infectious mortality pathways (42, 44). Surveillance and intervention strategies may therefore benefit from integrated approaches spanning CKD prevention and progression control, cardiometabolic risk management, infection prevention (including vaccination strategies) among dialysis and advanced CKD populations, and equitable access to kidney replacement therapies and transplantation (43, 45). The elevation during 2020–2023 should also be interpreted in the context of the COVID-19 pandemic. Patients with advanced CKD or kidney failure are highly vulnerable to infection-related complications, and dialysis care requires repeated healthcare contact, potentially increasing exposure risk. Pandemic-related disruptions may also have affected dialysis access, outpatient nephrology care, cardiovascular and metabolic risk management, and timely management of infections. In mortality certification, COVID-19 or other acute infectious conditions may have been selected as the underlying cause, while ESKD was recorded as a contributing condition. Therefore, the COVID-19-era increase in ESKD-involved mortality may reflect both true excess risk among ESKD populations and changes in underlying-cause attribution or documentation during the pandemic period.

This study leverages key strengths of U.S. vital statistics, including national coverage, standardized coding, and the availability of multiple-cause fields that enable any-mention ascertainment (46). Joinpoint regression provides an interpretable summary of non-linear temporal change using APC/AAPC metrics suited for public health surveillance (47). Use of ICD-10 chapter groupings supports cross-year comparability for primary structural analyses, while the NCHS 113 selected-cause list offers etiologic granularity for complementary assessments. Finally, estimation of *P_g,t_* links ESKD-involved deaths to the broader mortality base within each underlying-cause category, enabling interpretation in terms of ESKD penetration into cause-specific mortality rather than absolute burden alone (48). Our study extends prior CDC WONDER analyses of kidney failure-related mortality by moving beyond overall mortality trends to examine the underlying-cause structure of ESKD-involved deaths, estimate cause-specific ESKD involvement proportions, and decompose changes in ESKD-involved deaths into scale and penetration components. This framework provides insight into how ESKD is embedded within broader fatal disease pathways, rather than only quantifying the overall mortality burden.

Several limitations should be considered. First, death certificate data are susceptible to misclassification and underreporting of comorbidities such as ESKD; consequently, observed temporal patterns may reflect shifts in certification, coding, and reporting practices as well as genuine epidemiologic change. As CDC WONDER mortality coding employs the WHO ICD-10 mortality classification instead of ICD-10-CM, we defined ESKD involvement using the available ICD-10 mortality subcategories (N18.0/N18.5), which may not correspond exactly with clinical ESKD coding; however, this method appropriately captures end-stage kidney disease as recorded on death certificates for multiple-cause-of-death surveillance. Our code-specific verification analysis showed that N18.0 was observed during 1999–2010, whereas N18.5 appeared from 2011 onward and remained the only observed ESKD-related code thereafter. This finding supports the use of both available mortality-code categories for full-period surveillance, while also indicating that the abrupt fluctuation around 2011–2013 should be interpreted cautiously as a coding transition rather than a purely biological change. Second, CDC WONDER’s suppression of small counts and the instability of rates derived from sparse data limit subgroup granularity and can introduce non-random missingness, potentially affecting visual displays like heatmaps and certain estimates, especially within smaller strata and in recent years. Suppressed or unavailable cells were treated as missing rather than zero, and *P_g,t_* heatmaps and decomposition analyses were restricted to ICD-10 chapter groups meeting the prespecified ≥90% temporal coverage criterion. Missing tiles in heatmaps therefore indicate suppressed or unavailable estimates and should not be interpreted as zero ESKD involvement. Third, race reporting standards evolved during the study period; we analyzed bridged-race files (1999–2020) and single-race files (2021–2023) separately, so race-specific trends should be interpreted within, not across, these distinct frameworks. Accordingly, race-specific Joinpoint analyses were restricted to the 1999–2020 bridged-race period, whereas 2021–2023 single-race estimates were summarized descriptively and were not combined with bridged-race estimates to derive continuous race-specific AAPCs. Fourth, the aggregated nature of WONDER data precluded assessment of individual-level risk factors or clinical characteristics, including dialysis modality, vascular access, transplant status, or ESKD duration. Finally, the COVID-19 era constituted a major disruption to mortality patterns and healthcare delivery, and pandemic-period practices for attributing underlying cause and reporting contributing conditions may have influenced both the number and composition of deaths involving ESKD. The elevation during 2020–2023 may reflect several overlapping mechanisms, including increased infection-related mortality among patients with advanced CKD or kidney failure, disruptions to routine nephrology care and dialysis delivery, delayed management of cardiometabolic complications, and changes in death-certificate attribution when COVID-19 or infection was selected as the underlying cause and ESKD was recorded as a contributing condition. Therefore, pandemic-era changes should be interpreted as reflecting both true mortality risk and possible shifts in certification and healthcare-system context.

ESKD-involved mortality increased substantially in the United States from 1999 to 2023, with important non-linearities and persistent disparities across population subgroups. The underlying-cause portfolio among ESKD-involved deaths shifted meaningfully over time, and ESKD penetration into cause-specific mortality varied widely and evolved across decades. Monitoring ESKD as a contributing cause provides essential information beyond underlying-cause mortality and supports integrated prevention strategies addressing CKD progression and its cardiometabolic and infectious complications.

## Data Availability

The original contributions presented in the study are included in the article/Supplementary material, further inquiries can be directed to the corresponding author.
