# Supplementary material for: Trends, underlying-cause structure, and penetration of end-stage kidney disease involvement in U.S. mortality, 1999–2023: a multiple-cause-of-death analysis
Source: Front Public Health. 2026 Jun 19;14:1831145. doi: 10.3389/fpubh.2026.1831145 (PMC13327943; doi:10.3389/fpubh.2026.1831145)
Supplement: Supplementary file 1 [file Data_Sheet_1.docx]

**Trends, underlying-cause structure, and penetration of end-stage kidney disease involvement in U.S. mortality, 1999–2023: a multiple-cause-of-death analysis**

Kaide Xia^1^, Junwen Wang^2^, Zefa Meng^3^, Jingwen Yan^3*^

^1^ Guiyang Children's Hospital, The Maternal and Child Health CareHospital of Guizhou Medical University, Guiyang, China.

^2^ Department of Psychosomatic Medicine, The Second People’s Hospital of Guiyang, Guiyang, China;

^3^ Department of Nephrology, Zhejiang Provincial People's Hospital Bijie Hospital (The First People's Hospital of Bijie), Bijie, China.

**Correspondence to:** Jingwen Yan. Zhejiang Provincial People's Hospital Bijie Hospital (The First People's Hospital of Bijie), 112 Guanghui Road, Bijie 551700, China. Email: yanjingwen163@163.com.

**Running head:** ESKD-involved mortality in U.S.

**Supplementary Table S1. Disease groups and corresponding abbreviations.**

| **Disease groups** | **Abbreviation** |
| --- | --- |
| Diseases of the blood and blood-forming organs and certain disorders involving the immune mechanism | Blood/Immune |
| Diseases of the circulatory system | Circulatory |
| Congenital malformations, deformations and chromosomal abnormalities | Congenital |
| Diseases of the digestive system | Digestive |
| Diseases of the ear and mastoid process | Ear/Mastoid |
| Endocrine, nutritional and metabolic diseases | Endocrine/Metab |
| External causes of morbidity and mortality | External causes |
| Diseases of the eye and adnexa | Eye/Adnexa |
| Diseases of the genitourinary system | Genitourinary |
| Certain infectious and parasitic diseases | Infectious |
| Diseases of the musculoskeletal system and connective tissue | MSK/Connective |
| Mental and behavioural disorders | Mental/behav |
| Neoplasms | Neoplasms |
| Diseases of the nervous system | Nervous |
| Certain conditions originating in the perinatal period | Perinatal |
| Pregnancy, childbirth and the puerperium | Pregnancy |
| Diseases of the respiratory system | Respiratory |
| Diseases of the skin and subcutaneous tissue | Skin/Subcut |
| Codes for special purposes | Special codes |
| Symptoms, signs and abnormal clinical and laboratory findings, not elsewhere classified | Symptoms/Signs |

**Supplementary Table S2. Code-specific verification of ESKD-involved mortality coding in CDC WONDER among U.S. adults, 1999–2023**

| **Year** | **N18.0 deaths** | **N18.0 AAMR (95% CI)** | **N18.5 deaths** | **N18.5 AAMR (95% CI)** | **Interpretation** |
| --- | --- | --- | --- | --- | --- |
| 1999 | 25,738 | 14.51 (14.33, 14.69) | 0 | Not observed | N18.0 coding period |
| 2000 | 24,532 | 13.69 (13.52, 13.86) | 0 | Not observed | N18.0 coding period |
| 2001 | 26,585 | 14.56 (14.39, 14.74) | 0 | Not observed | N18.0 coding period |
| 2002 | 28,224 | 15.22 (15.04, 15.39) | 0 | Not observed | N18.0 coding period |
| 2003 | 29,548 | 15.67 (15.49, 15.85) | 0 | Not observed | N18.0 coding period |
| 2004 | 30,293 | 15.80 (15.63, 15.98) | 0 | Not observed | N18.0 coding period |
| 2005 | 30,996 | 15.91 (15.73, 16.09) | 0 | Not observed | N18.0 coding period |
| 2006 | 33,371 | 16.83 (16.65, 17.01) | 0 | Not observed | N18.0 coding period |
| 2007 | 33,871 | 16.73 (16.55, 16.91) | 0 | Not observed | N18.0 coding period |
| 2008 | 34,962 | 16.96 (16.78, 17.14) | 0 | Not observed | N18.0 coding period |
| 2009 | 36,351 | 17.27 (17.09, 17.45) | 0 | Not observed | N18.0 coding period |
| 2010 | 37,829 | 17.70 (17.52, 17.88) | 0 | Not observed | N18.0 coding period |
| 2011 | 0 | Not observed | 54,429 | 24.84 (24.63, 25.05) | Coding transition |
| 2012 | 0 | Not observed | 55,931 | 24.91 (24.70, 25.12) | Coding transition |
| 2013 | 0 | Not observed | 36,582 | 15.91 (15.75, 16.08) | N18.5 coding period |
| 2014 | 0 | Not observed | 38,640 | 16.42 (16.26, 16.59) | N18.5 coding period |
| 2015 | 0 | Not observed | 41,772 | 17.37 (17.20, 17.54) | N18.5 coding period |
| 2016 | 0 | Not observed | 44,195 | 18.02 (17.85, 18.19) | N18.5 coding period |
| 2017 | 0 | Not observed | 46,067 | 18.28 (18.11, 18.45) | N18.5 coding period |
| 2018 | 0 | Not observed | 48,354 | 18.82 (18.65, 18.99) | N18.5 coding period |
| 2019 | 0 | Not observed | 49,644 | 18.94 (18.78, 19.11) | N18.5 coding period |
| 2020 | 0 | Not observed | 56,065 | 21.01 (20.83, 21.18) | N18.5 coding period |
| 2021 | 0 | Not observed | 57,433 | 21.84 (21.66, 22.03) | N18.5 coding period |
| 2022 | 0 | Not observed | 55,070 | 20.23 (20.06, 20.40) | N18.5 coding period |
| 2023 | 0 | Not observed | 51,773 | 18.74 (18.58, 18.91) | N18.5 coding period |

**Abbreviations:** AAMR, age-adjusted mortality rate; CDC WONDER, Centers for Disease Control and Prevention Wide-ranging Online Data for Epidemiologic Research; CI, confidence interval; ESKD, end-stage kidney disease.

**Note:** Estimates were derived from CDC WONDER Multiple Cause of Death files among adults aged ≥25 years using the 2000 U.S. standard population. N18.0 was observed during 1999–2010 but not after 2010, whereas N18.5 was not observed during 1999–2010 and became the only observed code from 2011 onward, including 2021–2023 in the single-race files. This code-specific pattern indicates a coding transition around 2011 and explains the apparent discontinuity in ESKD-involved mortality trends around 2011–2013.

**Supplementary Table S3. Descriptive race-specific ESKD-involved mortality estimates from the CDC WONDER single-race files, 2021–2023**

| **Race** | **Year** | **Deaths, n** | **AAMR per 100,000 population (95% CI)** |
| --- | --- | --- | --- |
| Black | 2021 | 14660 | 52.87 (51.98, 53.75) |
| Black | 2022 | 13820 | 48.77 (47.94, 49.61) |
| Black | 2023 | 13117 | 45.33 (44.53, 46.12) |
| Other | 2021 | 3587 | 18.85 (18.22, 19.47) |
| Other | 2022 | 3693 | 18.29 (17.69, 18.89) |
| Other | 2023 | 3475 | 16.56 (16.00, 17.12) |
| White | 2021 | 39186 | 18.10 (17.92, 18.29) |
| White | 2022 | 37557 | 16.71 (16.54, 16.89) |
| White | 2023 | 35181 | 15.48 (15.32, 15.65) |

**Supplementary Table S4. Temporal coverage of** *P_g,t_* **estimates by ICD-10 chapter, 1999–2023**

| **ICD-10 chapter** | **Abbreviation** | **Available years, n** | **Total years, n** | **Temporal coverage, %** | **Missing years, n** | **Missing years** | **Included in *P_g,t_* heatmap** | **Included in decomposition analysis** | **Reason for exclusion if not included** |
| --- | --- | --- | --- | --- | --- | --- | --- | --- | --- |
| Diseases of the blood and blood-forming organs and certain disorders involving the immune mechanism | Blood/Immune | 25 | 25 | 100 | 0 | None | Yes | Yes | Not applicable |
| Diseases of the circulatory system | Circulatory | 25 | 25 | 100 | 0 | None | Yes | Yes | Not applicable |
| Congenital malformations, deformations and chromosomal abnormalities | Congenital | 25 | 25 | 100 | 0 | None | Yes | Yes | Not applicable |
| Diseases of the digestive system | Digestive | 25 | 25 | 100 | 0 | None | Yes | Yes | Not applicable |
| Endocrine, nutritional and metabolic diseases | Endocrine/Metab | 25 | 25 | 100 | 0 | None | Yes | Yes | Not applicable |
| External causes of morbidity and mortality | External causes | 25 | 25 | 100 | 0 | None | Yes | Yes | Not applicable |
| Diseases of the genitourinary system | Genitourinary | 25 | 25 | 100 | 0 | None | Yes | Yes | Not applicable |
| Certain infectious and parasitic diseases | Infectious | 25 | 25 | 100 | 0 | None | Yes | Yes | Not applicable |
| Diseases of the musculoskeletal system and connective tissue | MSK/Connective | 25 | 25 | 100 | 0 | None | Yes | Yes | Not applicable |
| Mental and behavioural disorders | Mental/behav | 25 | 25 | 100 | 0 | None | Yes | Yes | Not applicable |
| Neoplasms | Neoplasms | 25 | 25 | 100 | 0 | None | Yes | Yes | Not applicable |
| Diseases of the nervous system | Nervous | 25 | 25 | 100 | 0 | None | Yes | Yes | Not applicable |
| Diseases of the respiratory system | Respiratory | 25 | 25 | 100 | 0 | None | Yes | Yes | Not applicable |
| Diseases of the skin and subcutaneous tissue | Skin/Subcut | 25 | 25 | 100 | 0 | None | Yes | Yes | Not applicable |
| Certain conditions originating in the perinatal period | Perinatal | 22 | 25 | 88 | 3 | 2014, 2018, 2022 | No | No | Temporal coverage <90% due to suppressed or unavailable CDC WONDER cells |
| Pregnancy, childbirth and the puerperium | Pregnancy | 18 | 25 | 72 | 7 | 2011, 2018, 2019, 2020, 2021, 2022, 2023 | No | No | Temporal coverage <90% due to suppressed or unavailable CDC WONDER cells |
| Diseases of the eye and adnexa | Eye/Adnexa | 16 | 25 | 64 | 9 | 2000, 2005, 2011, 2013, 2014, 2015, 2018, 2019, 2020 | No | No | Temporal coverage <90% due to suppressed or unavailable CDC WONDER cells |
| Symptoms, signs and abnormal clinical and laboratory findings, not elsewhere classified | Symptoms/Signs | 15 | 25 | 60 | 10 | 1999, 2000, 2008, 2010, 2012, 2014, 2015, 2018, 2021, 2022 | No | No | Temporal coverage <90% due to suppressed or unavailable CDC WONDER cells |
| Diseases of the ear and mastoid process | Ear/Mastoid | 9 | 25 | 36 | 16 | 2000, 2003, 2005, 2007, 2009, 2010, 2011, 2012, 2014, 2016, 2017, 2018, 2019, 2021, 2022, 2023 | No | No | Temporal coverage <90% due to suppressed or unavailable CDC WONDER cells |
| Codes for special purposes | Special codes | 6 | 25 | 24 | 19 | 1999, 2000, 2002, 2003, 2004, 2005, 2006, 2007, 2008, 2009, 2010, 2011, 2012, 2013, 2014, 2015, 2016, 2017, 2018 | No | No | Temporal coverage <90% due to suppressed or unavailable CDC WONDER cells |

**Abbreviations:** CDC WONDER, Centers for Disease Control and Prevention Wide-ranging Online Data for Epidemiologic Research; ESKD, end-stage kidney disease; *P_g,t_*, ESKD involvement proportion.

**Note:** *P_g,t_* estimates were calculated as the number of ESKD-involved deaths within each ICD-10 chapter divided by all deaths with the same underlying-cause ICD-10 chapter. Suppressed or unavailable CDC WONDER year–cause cells were treated as missing rather than zero. ICD-10 chapters with *P_g,t_* estimates available for at least 90% of years in the 1999–2023 analysis window were included in the primary *P_g,t_* heatmap and decomposition analyses.


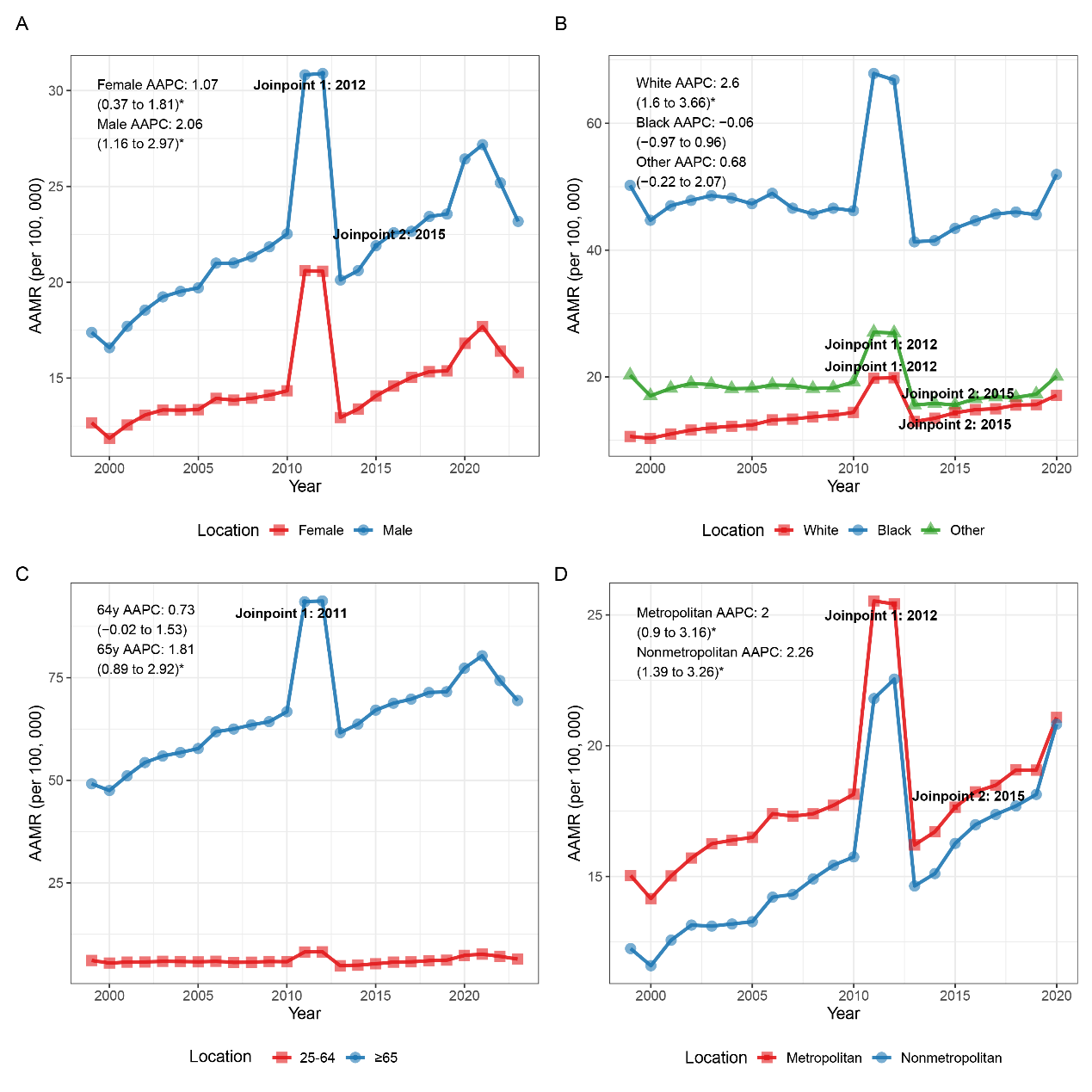


**Supplementary Figure S1. Joinpoint trends in ESKD-involved AAMR by sex, race, age, and urbanization.** Sex and age trends are shown for 1999–2023; race-specific trends are restricted to the 1999–2020 bridged-race files; urbanization trends are shown for 1999–2020. AAMR, age-adjusted mortality rate; ESKD, end-stage kidney disease.
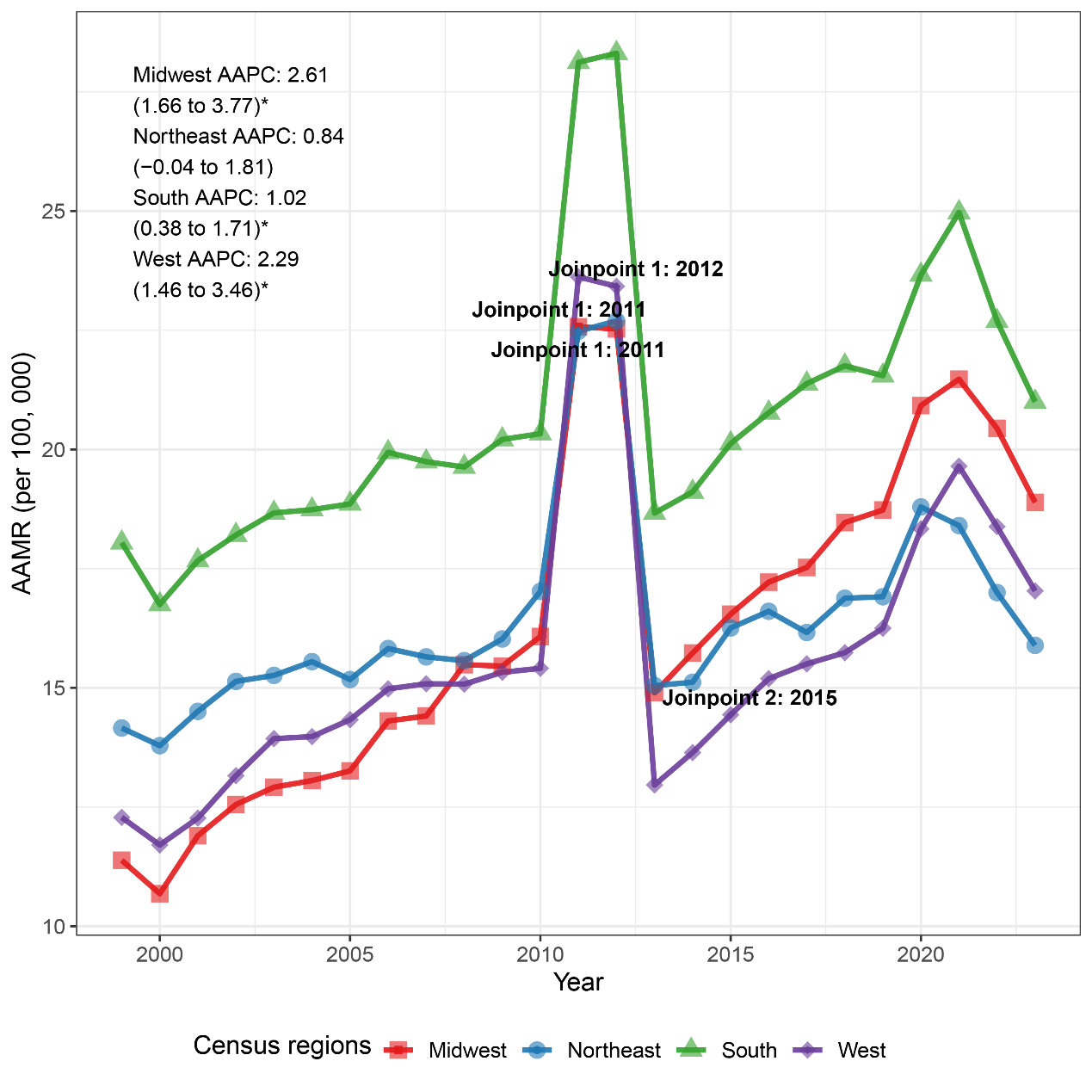


**Supplementary Figure S2. Joinpoint trends in ESKD-involved AAMR by U.S. census region, 1999–2023.** AAMR, age-adjusted mortality rate; ESKD, end-stage kidney disease.


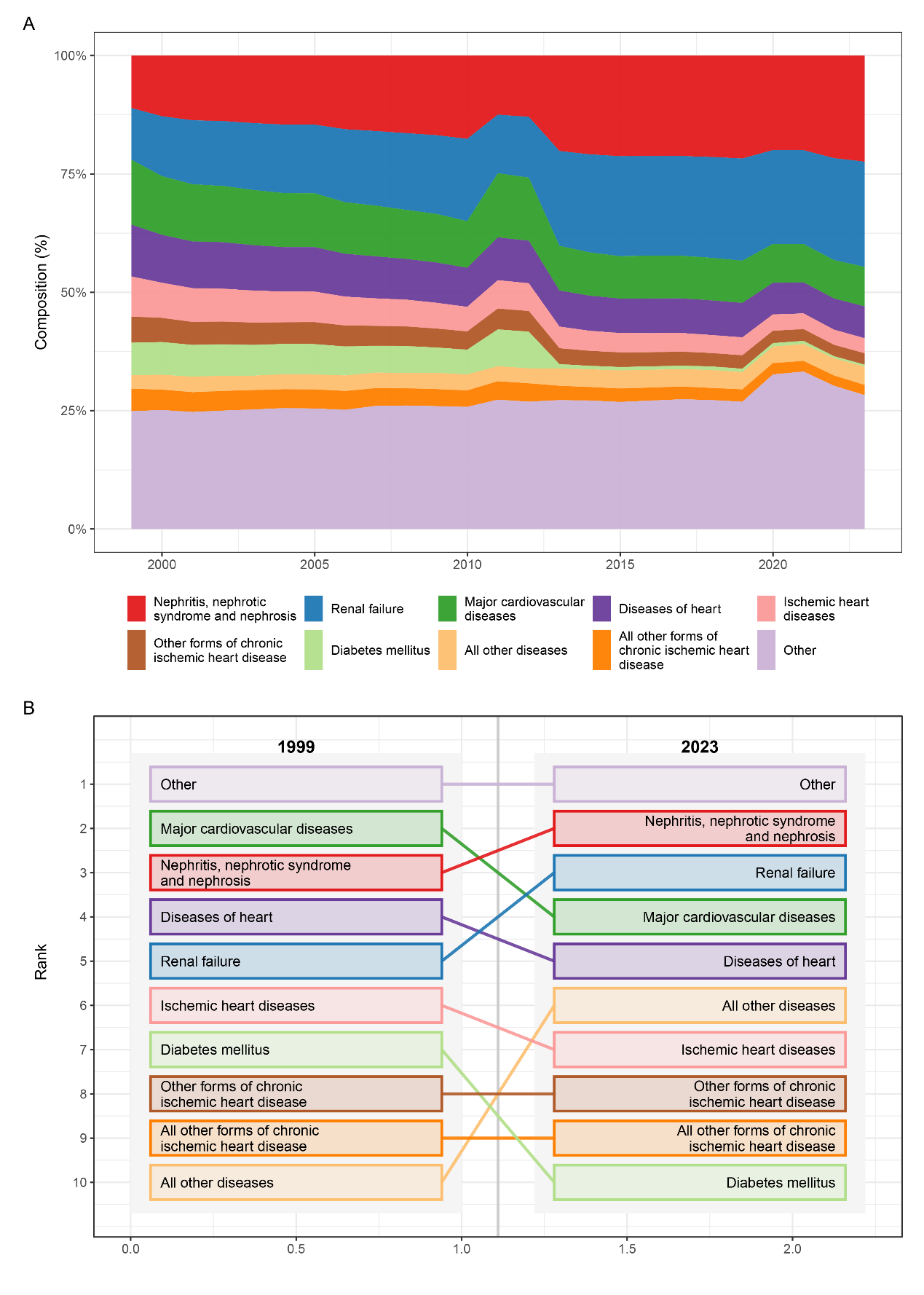


**Supplementary Figure S3. Underlying-cause composition and rank shifts of ESKD-involved deaths using the NCHS 113 selected-cause list, United States, 1999–2023.** ESKD, end-stage kidney disease; NCHS, National Center for Health Statistics.


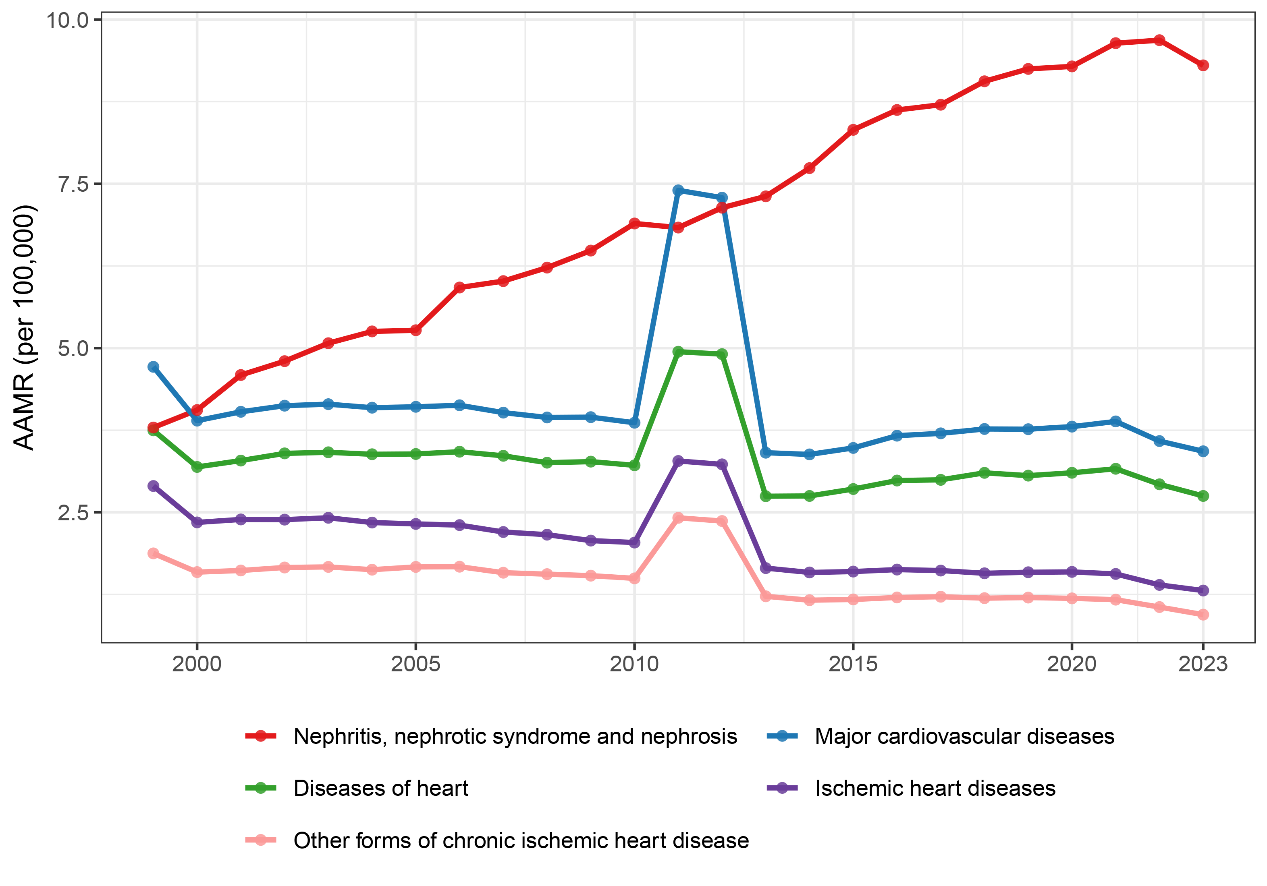


**Supplementary Figure S4. AAMR trends for the top five NCHS 113 underlying-cause categories among ESKD-involved deaths, United States, 1999–2023.** AAMR, age-adjusted mortality rate; ESKD, end-stage kidney disease; NCHS, National Center for Health Statistics.

**
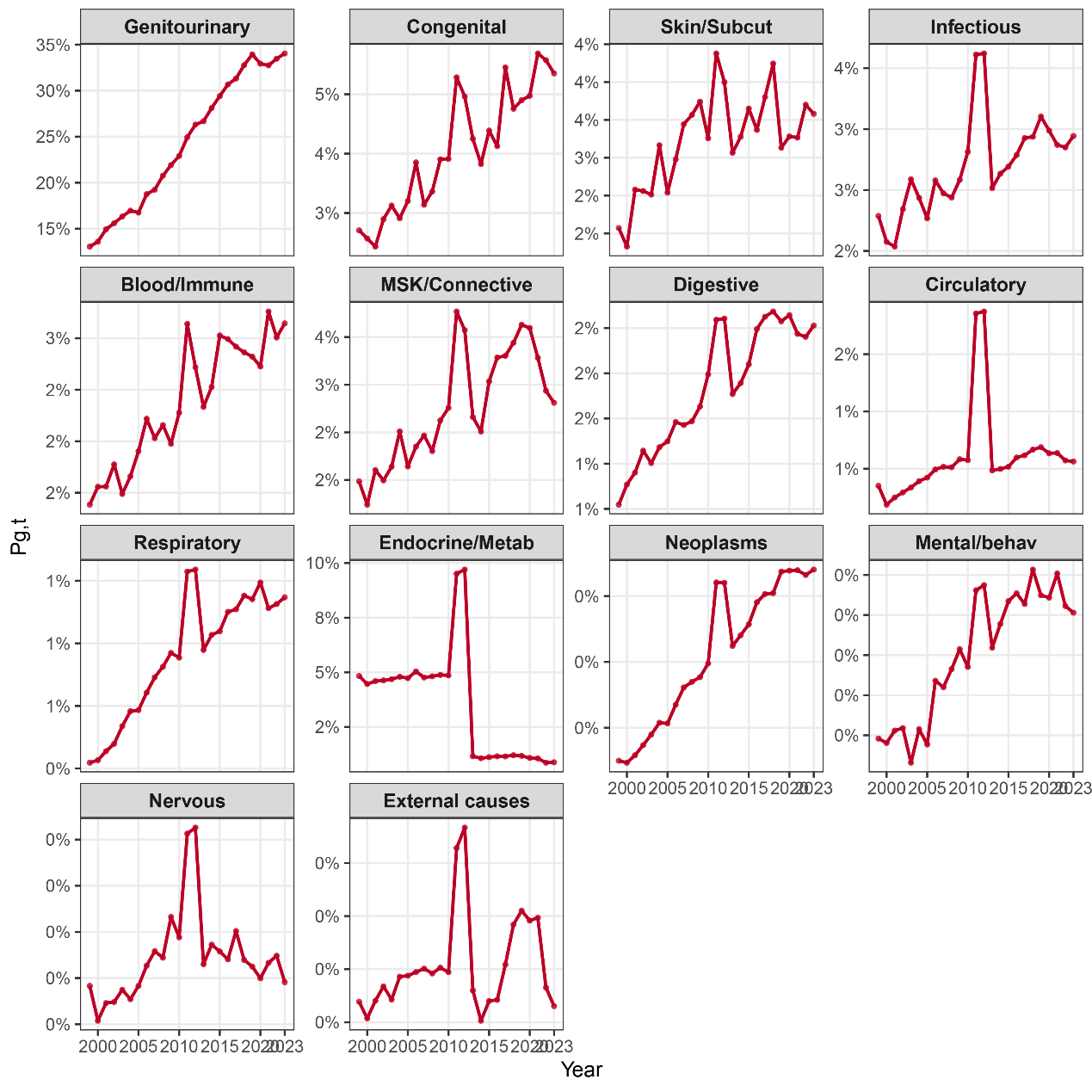
**

**Supplementary Figure S5. Temporal trends in the ESKD involvement proportion (*P_g,t_*) across ICD-10 chapter–defined underlying causes, United States, 1999–2023.** *P_g,t_* was defined as the proportion of deaths within a given underlying-cause ICD-10 chapter in which ESKD was also recorded anywhere on the death certificate. ICD-10, International Classification of Diseases, 10th Revision; ESKD, end-stage kidney disease.

**
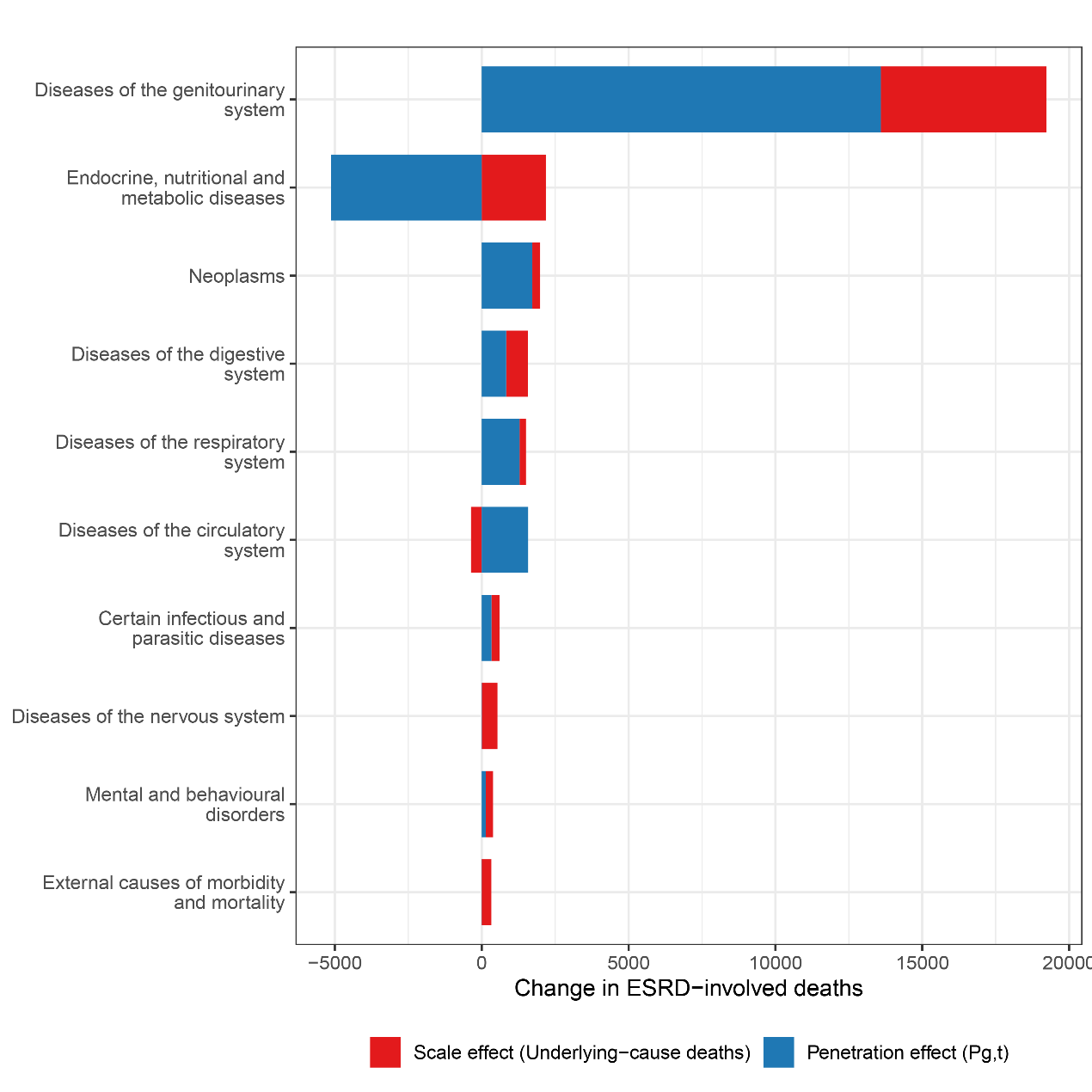
**

**Supplementary Figure S6. Decomposition of changes in ESKD-involved deaths into scale and penetration components across ICD-10 chapters, United States, 1999–2023.** International Classification of Diseases, 10th Revision; ESKD, end-stage kidney disease.

**
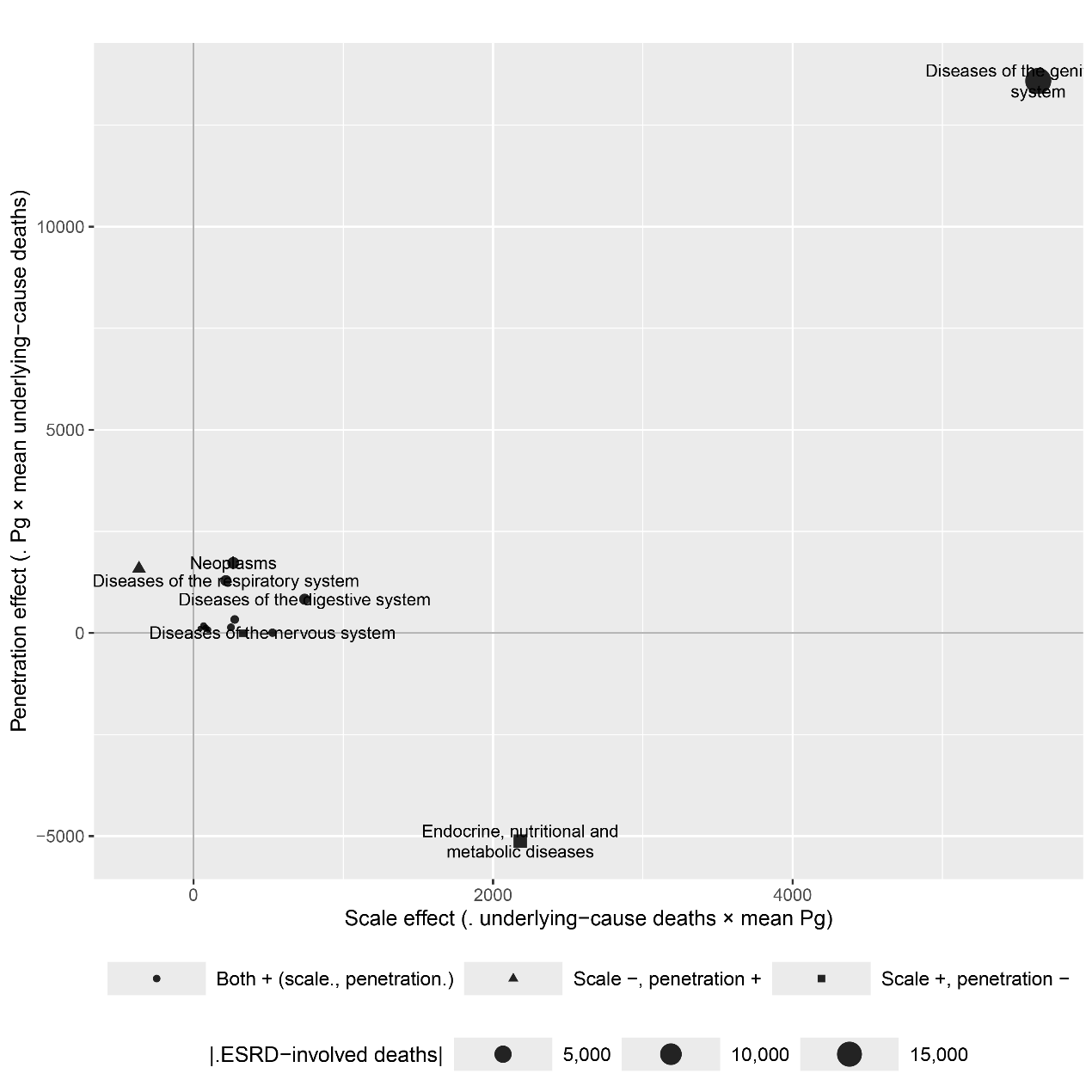
**

**Supplementary Figure S7. Scale–penetration driver space for ESKD-involved deaths by ICD-10 chapter, United States, 1999–2023.** International Classification of Diseases, 10th Revision; ESKD, end-stage kidney disease.

**
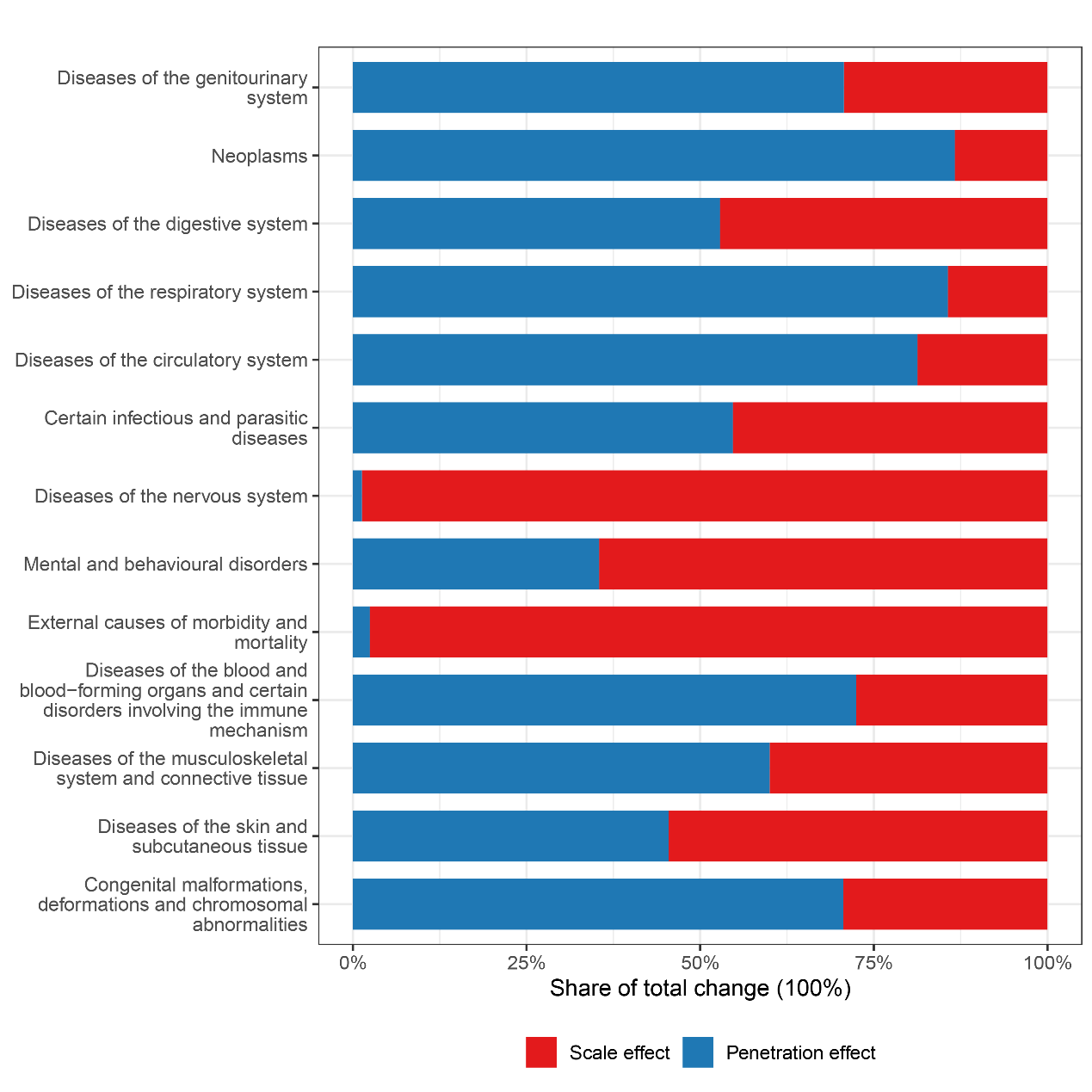
 Supplementary Figure S8. Relative contributions of scale versus penetration to the total change in ESKD-involved deaths by ICD-10 chapter, United States, 1999–2023.** ICD-10, International Classification of Diseases, 10th Revision; ESKD, end-stage kidney disease.

**
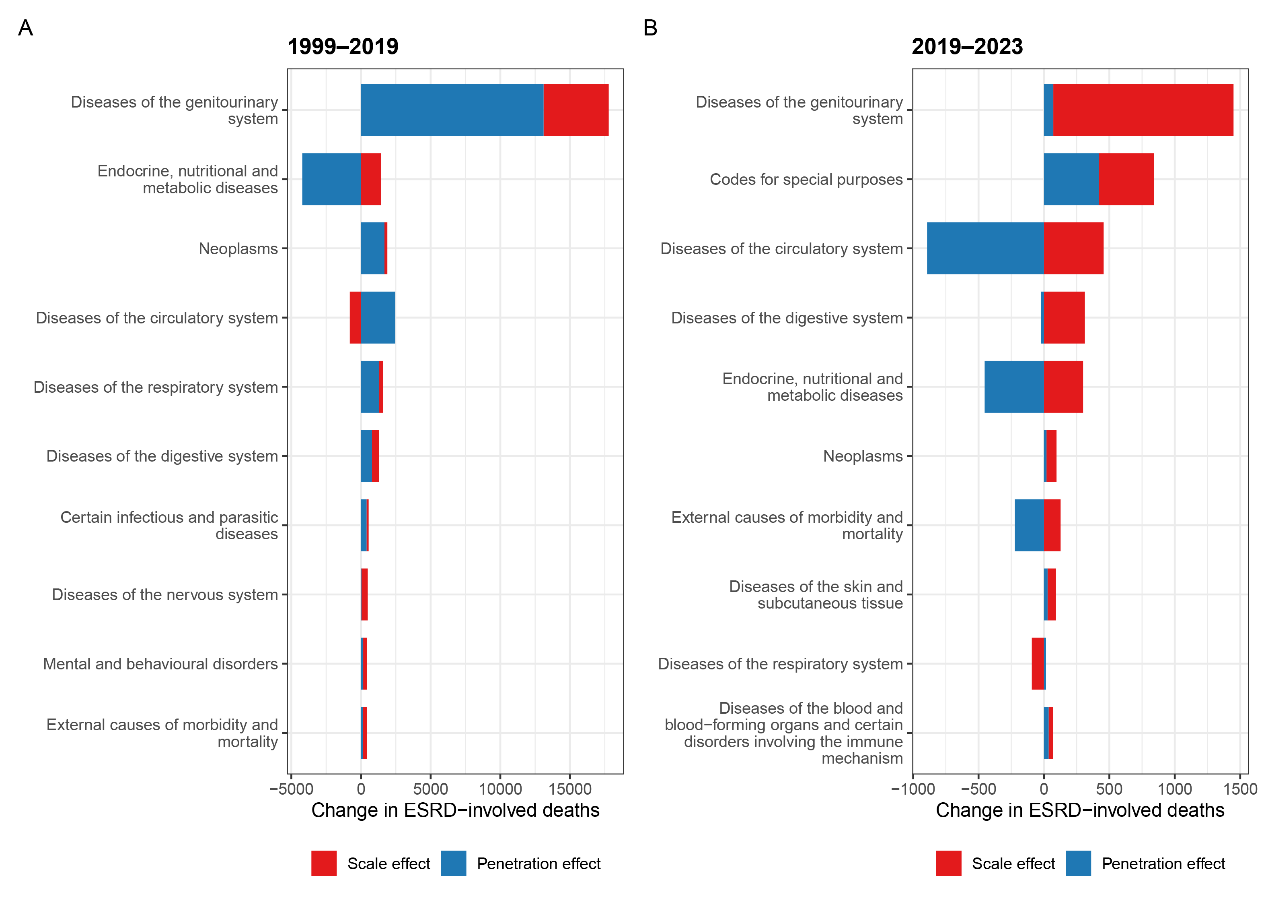
**

**Supplementary Figure S9. Period-specific decomposition of ESKD-involved deaths by ICD-10 chapter: 1999–2019 versus 2019–2023.** International Classification of Diseases, 10th Revision; ESKD, end-stage kidney disease.
